# Bioinformatics analysis of differentially expressed genes and pathways in the development of cervical cancer

**DOI:** 10.1186/s12885-021-08412-4

**Published:** 2021-06-26

**Authors:** Baojie Wu, Shuyi Xi

**Affiliations:** Shanghai Zerun Biotechnology Co., Ltd., Pilot Department, Building 9, 1690 Zhangheng Road Pudong, Shanghai, 201203 China

**Keywords:** Bioinformatics analysis, Cervical intraepithelial neoplasia, Cervical cancer, Differentially expressed genes, Functional enrichments

## Abstract

**Background:**

This study aimed to explore and identify key genes and signaling pathways that contribute to the progression of cervical cancer to improve prognosis.

**Methods:**

Three gene expression profiles (GSE63514, GSE64217 and GSE138080) were screened and downloaded from the Gene Expression Omnibus database (GEO). Differentially expressed genes (DEGs) were screened using the GEO2R and Venn diagram tools. Then, Gene Ontology (GO) and Kyoto Encyclopedia of Genes and Genomes (KEGG) pathway enrichment analyses were performed. Gene set enrichment analysis (GSEA) was performed to analyze the three gene expression profiles. Moreover, a protein–protein interaction (PPI) network of the DEGs was constructed, and functional enrichment analysis was performed. On this basis, hub genes from critical PPI subnetworks were explored with Cytoscape software. The expression of these genes in tumors was verified, and survival analysis of potential prognostic genes from critical subnetworks was conducted. Functional annotation, multiple gene comparison and dimensionality reduction in candidate genes indicated the clinical significance of potential targets.

**Results:**

A total of 476 DEGs were screened: 253 upregulated genes and 223 downregulated genes. DEGs were enriched in 22 biological processes, 16 cellular components and 9 molecular functions in precancerous lesions and cervical cancer. DEGs were mainly enriched in 10 KEGG pathways. Through intersection analysis and data mining, 3 key KEGG pathways and related core genes were revealed by GSEA. Moreover, a PPI network of 476 DEGs was constructed, hub genes from 12 critical subnetworks were explored, and a total of 14 potential molecular targets were obtained.

**Conclusions:**

These findings promote the understanding of the molecular mechanism of and clinically related molecular targets for cervical cancer.

**Supplementary Information:**

The online version contains supplementary material available at 10.1186/s12885-021-08412-4.

## Background

Human papillomavirus (HPV) infection is a primary cause of cervical cancer and led to 311,365 deaths in 2018 [1]. Cervical intraepithelial neoplasia (CIN) is a potentially premalignant transformation of squamous cells of the cervix. From normal to CIN (N-CIN) and ultimately to cancer (CIN-CC), cervical cancer is a continuous and evolving process [2, 3]. In addition to HPV vaccination, early diagnosis and treatment can reduce the mortality rate of cervical cancer. In recent years, the prognosis of cervical cancer has been a concern. The identification of biomarkers correlated with the diagnosis and prognosis of cervical cancer is of great importance. With the development of bioinformatics, more studies have focused on the signaling and metabolic pathways of cervical cancer, data mining and validation of related biomolecular targets. The aim of this study was to explore and identify the key genes and signaling pathways contributing to the progression of cervical cancer to improve prognosis. An integrated bioinformatics analysis was performed to select differentially expressed genes (DEGs) and hub genes and to investigate their protein–protein interaction (PPI) networks, related prognostic signatures, functional annotations and potential prognostic value. This study may offer better insight into potential molecular mechanisms to explore preventive and therapeutic strategies.

## Methods

### Data processing

CIN is an important process in the development of cervical cancer. The gene expression profiles related to CIN progression were retrieved and downloaded from the Gene Expression Omnibus (GEO) database of the National Center for Biotechnology Information (NCBI). The retrieval formula was as follows: (“cervical intraepithelial neoplasia” [MeSH Terms] OR cervical intraepithelial neoplasia [All Fields]) AND “*Homo sapiens*” [porgn] AND (“Expression profiling by array” [Filter] AND (“0001/01/01” [PDAT]: “2020/11/27” [PDAT])). Three expression profile microarray datasets (GSE63514, GSE64217 and GSE138080) were selected and downloaded from the GEO database for analysis.

**Fig. 1:**
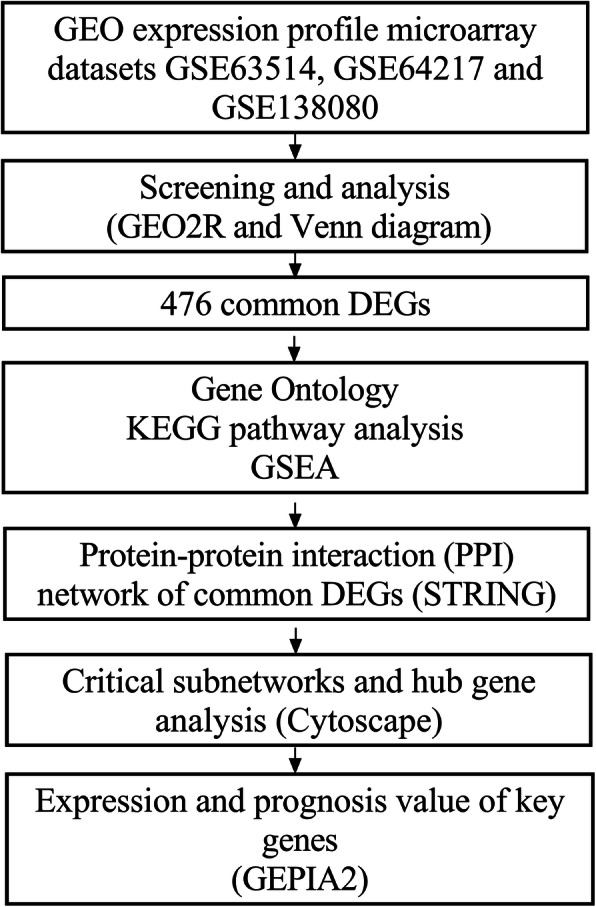
Flowchart of the integrated analysis

GSE63514 [4] is an expression profile based on the GPL570 platform (Affymetrix Human Genome U133 Plus 2.0 Array) and contains samples of normal cervical epithelium, CIN and cervical squamous epithelial cancer. GSE64217 is an expression profile based on the GPL10558 platform (Illumina HumanHT-12 V4.0 expression beadchip), and GSE64217 was provided by the Indian Institute of Technology Kharagpur, School of Medical Science and Technology, Multimodal Imaging and Computing for Theranostics. It contains samples of normal cervical mucosa, CIN and cervical squamous cell carcinoma (CESC). GSE138080 [5] is an expression profile based on the GPL4133 platform (Agilent-014850 Whole Human Genome Microarray 4x44K G4112F) and contains samples of normal cervical squamous epithelium, CIN and CESC. Samples of the three microarray expression profiling datasets were classified and analyzed according to the progression of cervical cancer. The workflow of this study is indicated in Fig. 1.

### Analysis of microarray datasets

According to the progression of cervical cancer, in the subsequent analysis, samples of each dataset were divided into three groups: N-CIN, CIN-CC, and N-CC. The GEO2R tool was used to analyze the three expression datasets [6, 7]. Normalization and log2 conversion were carried out for each dataset to filter out the DEGs of the three datasets, and the DEGs are displayed as volcano plots. The filtering conditions were as follows: |log2-fold change| ≥ 1 and adjusted *P-*value (adj. *P*) < 0.05. Then, the Venn diagram tool (http://bioinformatics.psb.ugent.be/webtools/Venn/) was used to compare and analyze the results of the intersection analysis. Based on the intersection of datasets, the final DEGs were obtained. In this study, we selected DEGs according to the intersection of at least two expression profile datasets to avoid the disadvantages of a single dataset and then integrated the results for further biological function analysis.

### Functions and pathways of DEGs

DAVID Bioinformatics Resources 6.8 was utilized to distinguish and enrich the biological attributes, such as biological processes, cellular components, molecular functions and pathways, of important DEGs [8] (https://david.ncifcrf.gov/). Moreover, Kyoto Encyclopedia of Genes and Genomes (KEGG) pathway [9] and Gene Ontology (GO) enrichment analyses were used to identify the significant pathways. *P* < 0.05 was set as the cutoff criterion for significant enrichment.

### Enrichment analysis of key pathways and core genes

Gene set enrichment analysis (GSEA) was used to determine the key pathways and core genes during the development of cervical cancer [10, 11]. Enrichment analyses were conducted to determine whether a series of a priori-defined biological processes were enriched. The enriched pathways were arranged in the order of their normalized enrichment scores, and those with *P* < 0.01 were chosen for further analysis. The results of the GSEA of different expression profile datasets were intersected to obtain the common significant KEGG pathways, and core gene sets were analyzed.

### Construction of the PPI network

The STRING database is an online search tool used to analyze known proteins and predict PPI networks, including direct and indirect interactions between proteins and their functional correlations [12] (https://string-db.org/). Molecular interactions and PPI networks can promote the exploration of molecular targets, signaling and metabolic pathways, and network functions involved in the progression of cervical lesions. Therefore, the STRING database was used to construct the PPI network.

### Critical subnetworks and hub genes

Hub genes play an important role in biological processes. Based on the PPI network, hub genes were screened according to network topology. Cytoscape software (version 3.8.2, cytoHubba and MCODE plug-ins) was used to discover the key targets or subnetworks of complex networks [13–15]. The critical subnetworks and hub genes during the development of cervical cancer were analyzed.

### Expression and prognostic value of hub genes

As cervical cancer is a complex disease, its etiopathogenesis involves compound gene expression and multiple interactions. GEPIA2 (http://gepia2.cancer-pku.cn/) was used to analyze the expression of multiple hub genes and the prognostic value of the selected hub genes in CESC [16]. GEPIA2 can be used to perform principal component analysis (PCA) of genes and presents results as 2D plots. To evaluate the potential clinical value of hub genes more comprehensively, PCA dimensionality reduction was performed. Multiple gene comparison in different cancer species also provided a reference for the prognostic evaluation of different genes in cervical lesions. Moreover, the functional enrichment of prognostic genes was demonstrated.

## Results

### Identification of DEGs

**Fig. 2:**
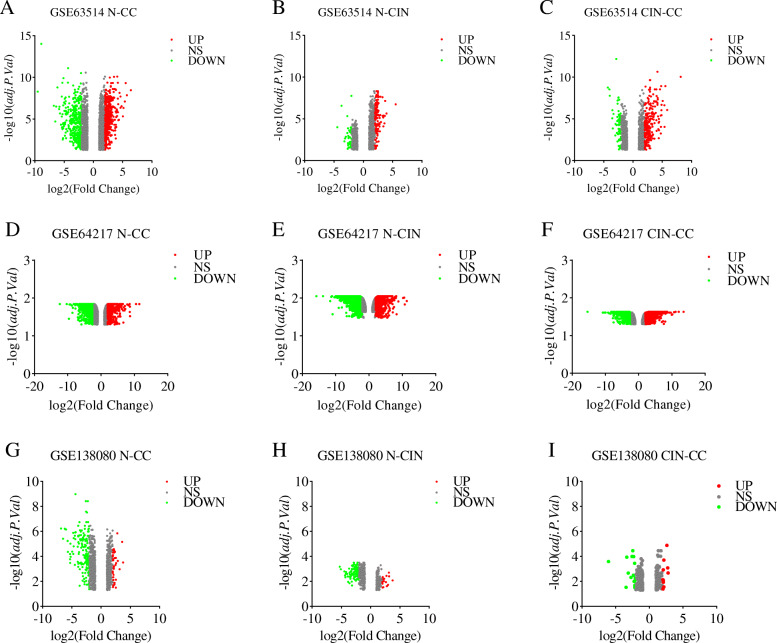
Correlations of all DEGs from three different expression profiling microarrays. **A** ~ **C**: GSE63514, **D** ~ **F**: GSE64217, **G** ~ **I**: GSE138080

**Fig. 3:**
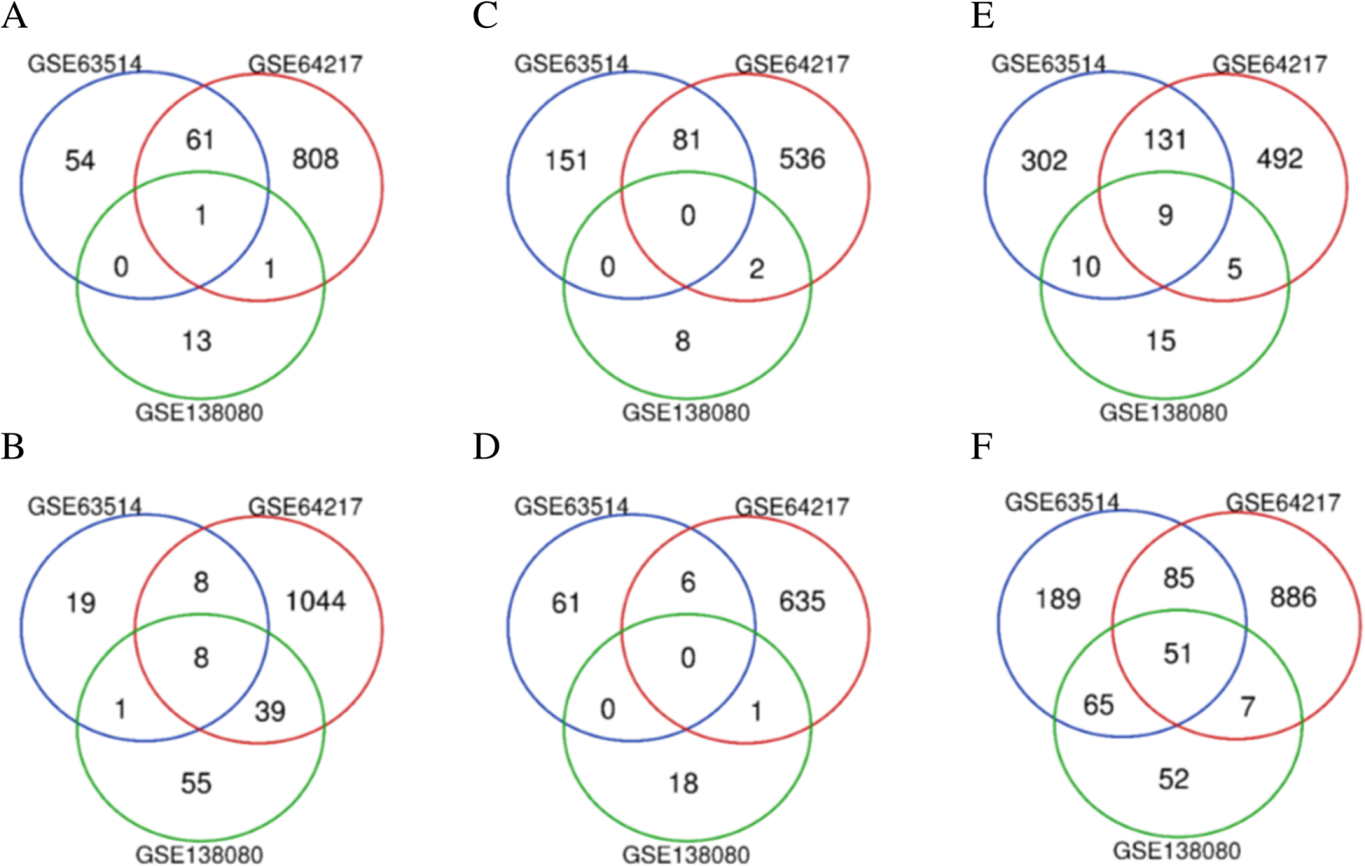
Screening DEGs via intersection analysis. Upregulated genes (**A**) and downregulated genes (**B**) in the N-CIN group. Upregulated genes (**C**) and downregulated genes (**D**) in the CIN-CC group. Upregulated genes (**E**) and downregulated genes (**F**) in the N-CC group

Volcano plots (Fig. 2) showed the correlation of all DEGs from the three expression profiling microarrays. After comprehensive analysis and screening with the Venn diagram tool (Fig. 3), 63 upregulated genes were identified in the N-CIN group, and *CDKN2A* [17, 18] was shared among the three datasets. Among the 56 downregulated genes, *EMP1*, *CRISP2*, *ALOX12*, *DMKN*, *ZBED2*, *PPP1R3C*, *CDA* and *CRCT1* were shared among the three datasets. There were 83 upregulated genes and 7 downregulated genes in the CIN-CC group. Furthermore, 155 upregulated genes in the N-CC group, including *TCAM1P*, *MCM2*, *HS6ST2*, *AIM2*, *CDKN2A*, *RFC4*, *PLOD2*, *APOC1* and *CENPF* [19], were shared among the three datasets. Among the 208 downregulated genes, 51 were shared among the three datasets: *TMPRSS11B*, *BBOX1*, *ZSCAN18*, *ENDOU*, *KLK8*, *ANKRD35*, *A2ML1*, *CRYAB*, *MAL*, *VSIG10L*, *ECM1*, *SPINK5*, *TM7SF2*, *SPINK7*, *PRSS27*, *RBP7*, *PRSS3*, *ACPP*, *HPGD*, *CWH43*, *RHCG*, *SCEL*, *TP53I3*, *SPRR2C*, *CRABP2*, *HCG22*, *DMKN*, *PRSS2*, *CLIC3*, *SPNS2*, *LCE3D*, *FUT3*, *RDH12*, *CRNN*, *CEACAM7*, *LYNX1*, *MYZAP*, *KRTDAP*, *NDRG4*, *SLC5A1*, *GPX3*, *PPP1R3C*, *SLURP1*, *SLC24A3*, *THSD4*, *PSCA*, *CDA*, *FAM3D*, *CFD*, *HOPX*, and *CRCT1*. Finally, after deletion of duplicate genes, a total of 476 DEGs were screened (|log2-fold change| ≥ 1 and adj. *P* < 0.05): 253 genes with upregulated expression and 223 genes with downregulated expression.

### GO and KEGG pathway enrichment analyses

A total of 476 DEGs were uploaded to DAVID for GO/KEGG analyses. The terms of each GO category are provided in Additional file 1: Table S1, Table S2 and Table S3. Most DEGs were enriched in the biological processes keratinization, epidermis development, DNA replication, mitotic division, cell cycle, proteolysis, regulation of cell proliferation, cell cycle, and related activity of enzymes; the cellular components extracellular space, cornified envelope, extracellular exosome, and extracellular region; and the molecular functions serine-type endopeptidase activity, serine-type peptidase activity, structural molecule activity, cysteine-type and endopeptidase inhibitor activity. The results of KEGG pathway enrichment are shown in Table 1.

**Table 1:** KEGG pathway enrichment analysis of DEGs (DAVID)

Term	Count	*P*-value
hsa04110: Cell cycle	14	7.05E-07
hsa03030: DNA replication	7	5.88E-05
hsa05323: Rheumatoid arthritis	8	0.001555896
hsa04115: p53 signaling pathway	7	0.001840686
hsa05219: Bladder cancer	5	0.007981073
hsa04610: Complement and coagulation cascades	6	0.01080063
hsa05146: Amoebiasis	7	0.017024795
hsa04060: Cytokine-cytokine receptor interaction	11	0.019861501
hsa04114: Oocyte meiosis	7	0.020898334
hsa00590: Arachidonic acid metabolism	5	0.030571729

### GSEA enrichment of expression datasets

**Fig. 4:**
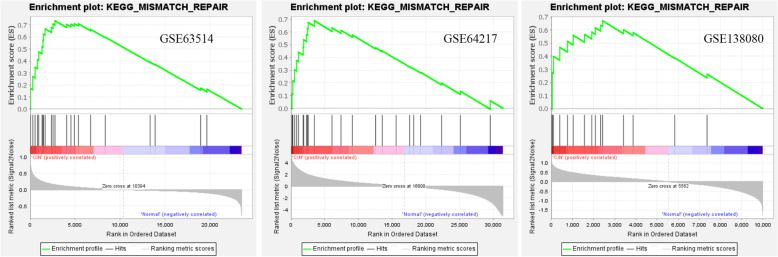
GSEA snapshots of KEGG pathway enrichment analysis: DNA mismatch repair (N-CIN)

**Fig. 5:**
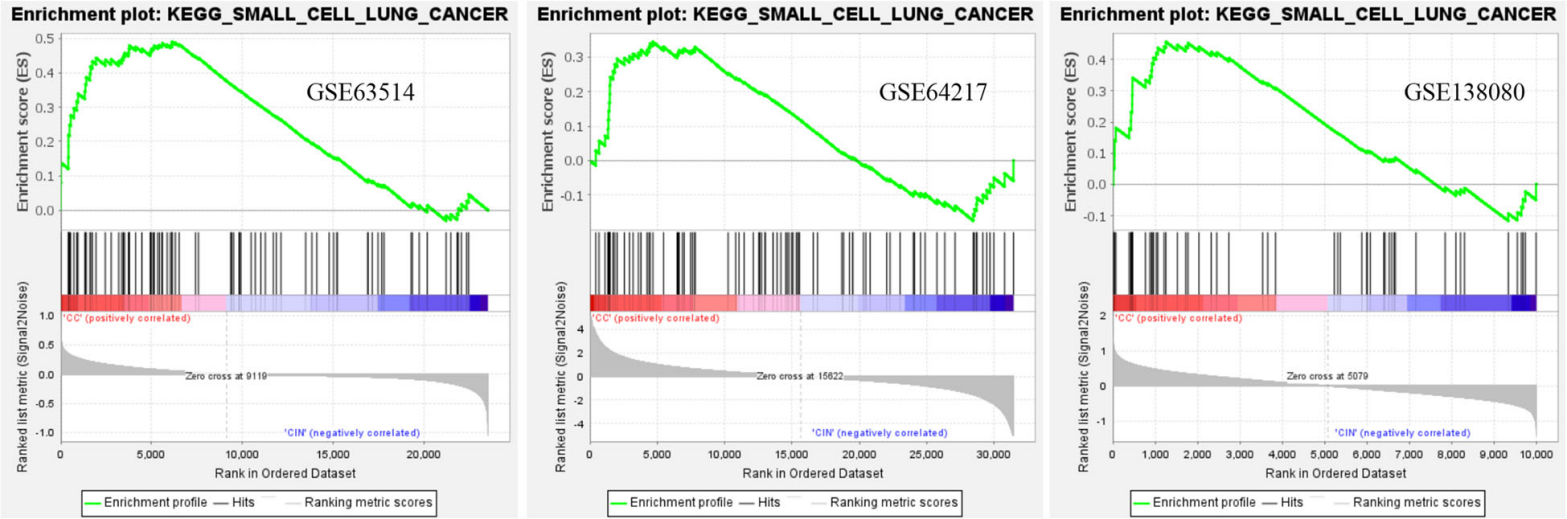
GSEA snapshots of KEGG pathway enrichment analysis: Small cell lung cancer (CIN-CC)

**Fig. 6:**
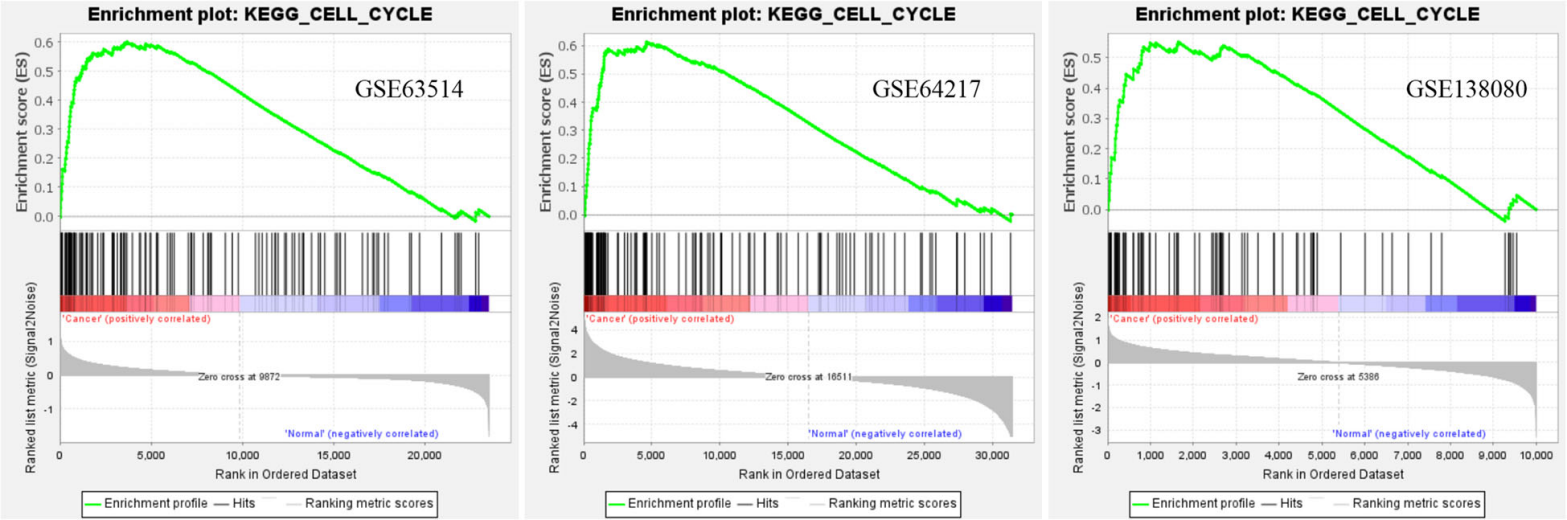
GSEA snapshots of KEGG pathway enrichment analysis: Cell cycle (N-CC)

Expression datasets (GSE63514, GSE64217 and GSE138080) were subjected to GSEA (version 4.1.0). Then, key pathways and core related genes were obtained. The results showed 5 common KEGG pathways in the N-CIN group (*p* < 0.01), namely, DNA mismatch repair (*PCNA* [20], *EXO1* [21], *POLD1*, *MSH6*, and *LIG1*), the cell cycle (*MCM3*, *MCM5* [22], *CDC6*, *MCM6* [23], *CHEK2*, *PKMYT1*, *CDC7*, *RBL1*, *WEE1*, *CCNA2* [24], and *TTK*), DNA replication (*PCNA*, *POLD1*, *DNA2*, *MCM3*, *MCM5*, *PRIM2*, *POLE2*, *MCM6*, *LIG1*, *RNAEH2A*, and *PRIM1*), cysteine and methionine metabolism (*LDHC*), and nucleoside exception repair (*PCNA*, *POLD1*, *POLE2*, and *LIG1*). In addition, there were 6 common KEGG pathways in the CIN-CC group (*p* < 0.01), namely, the adipocytokine signaling pathway, small cell lung cancer (*ITGA6* [25], *PIAS3*, and *LAMC2* [26]), pathways in cancer, the Toll-like receptor signaling pathway, graft versus host disease, and the TGF-beta signaling pathway [27]. There were 9 common KEGG pathways in the N-CC group (*p* < 0.01), namely, the cell cycle (*PCNA*, *CDC25B*, *MCM3*, *MCM5*, *CDC6* [28], *GSK3B*, *MCM6*, *CHEK2*, *PKMYT1*, *CDC20* [29], *PTTG1*, *SMAD3*, *CCNB1* [30], *RBL1*, *CDC7* [23], *WEE1*, *CDK2* [31], *CCNA2*, and *TTK*), nucleotide excision repair, the Toll-like receptor signaling pathway, prion diseases, spliceosome, DNA replication, proteasome, colorectal cancer, and pancreatic cancer. Moreover, GSEA showed that DNA mismatch repair (N-CIN), small cell lung cancer (CIN-CC), and the cell cycle (N-CC) were the most significantly enriched pathways (*P* < 0.01, FDR < 0.05), and snapshots of the enrichment analysis are shown in Figs. 4, 5 and 6.

### PPI network construction and hub genes of cervical lesions

**Fig. 7:**
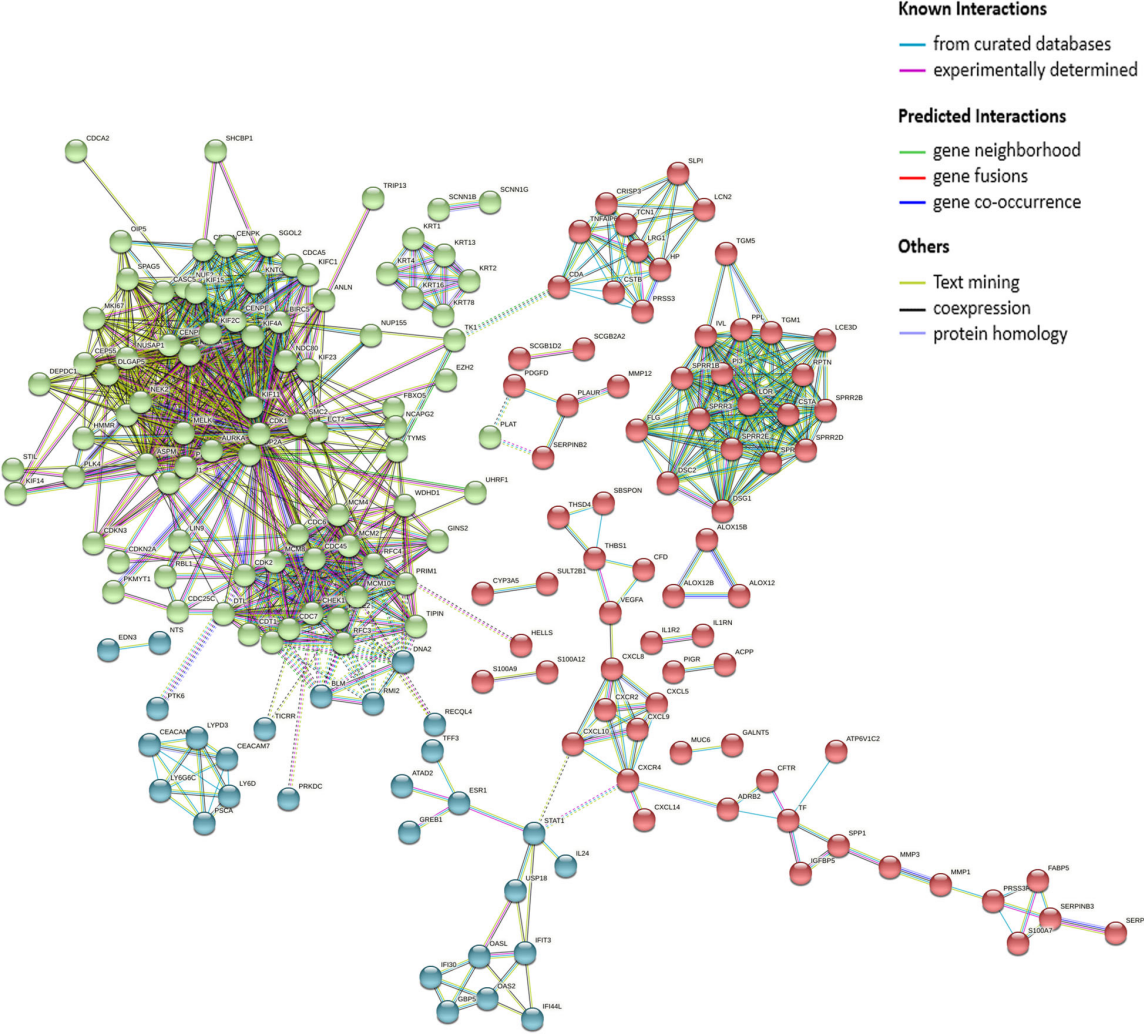
PPI network of the DEGs

**Fig. 8:**
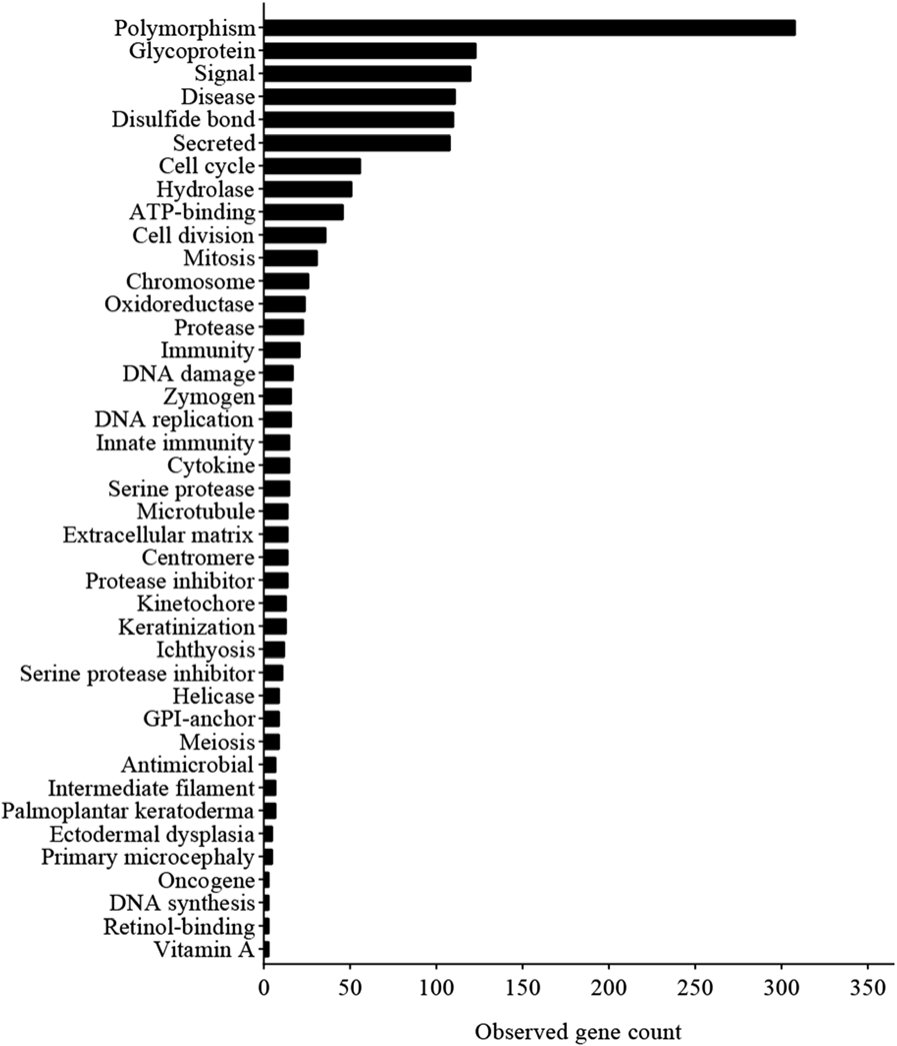
Functional enrichment in the PPI network of DEGs

A total of 476 DEGs were uploaded to the STRING database to construct the PPI network (minimum required interaction score: highest confidence 0.900, k-means clustering: number of clusters 3). The PPI network is shown in Fig. 7. There were 415 nodes and 931 edges in the network (PPI enrichment *P* < 1.0E-16). The functional enrichment analysis in the PPI network included 387 GO terms, 5 KEGG pathways, 64 Reactome pathways, and 111 protein domains. Significantly enriched functions of the network are shown in Fig. 8. According to these results, most of the proteins were distributed among the following aspects: polymorphism, glycoprotein, signal, disease, disabled bond, and secreted. These genes express proteins and then interact functionally in the PPI network, revealing their role in the progression of cervical cancer.

**Fig. 9:**
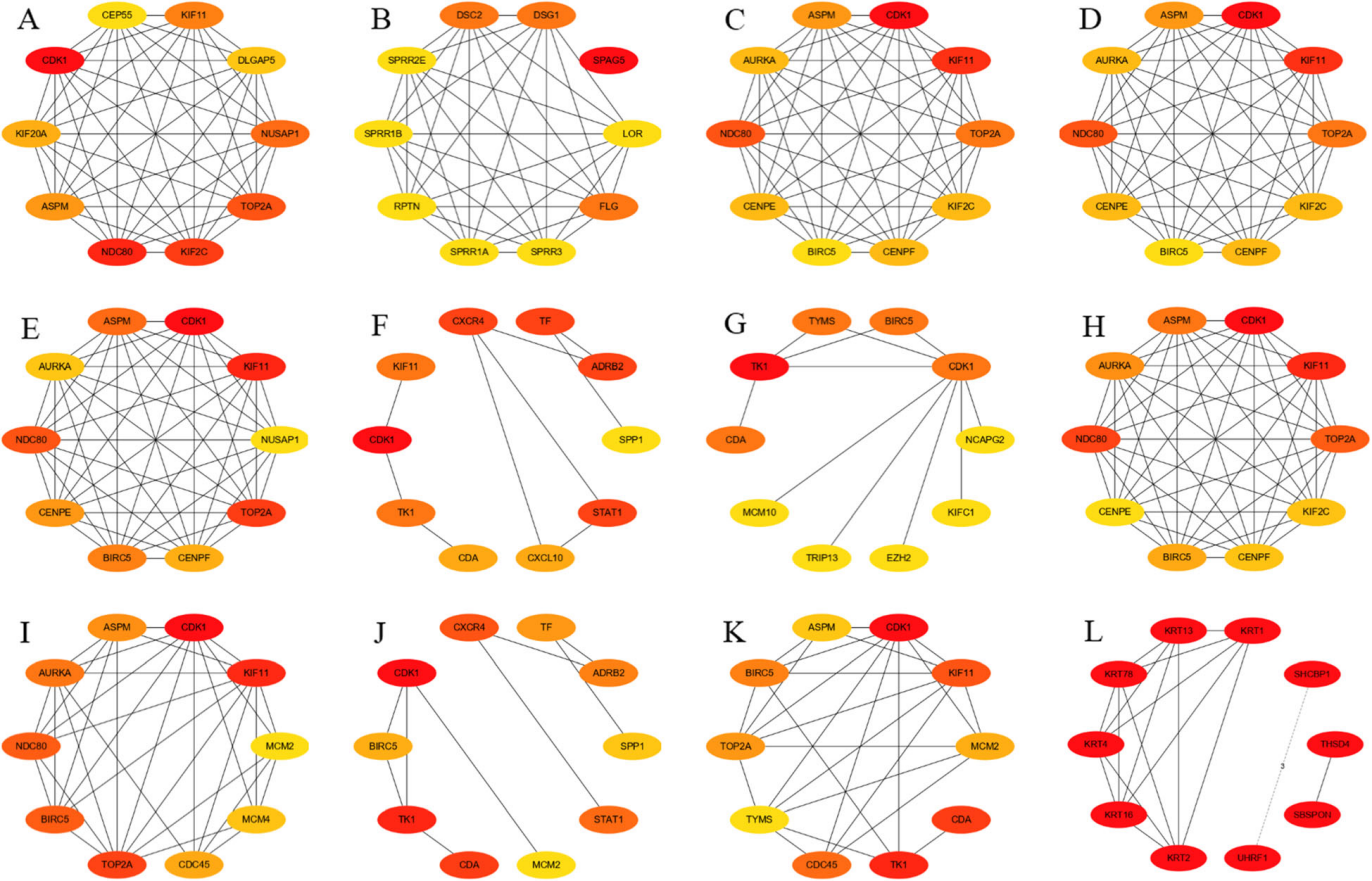
Discovering high scoring hub genes in cervical cancer development (cytoHubba). Topological analysis: (**A**) MCC, (**B**) DMNC, (**C**) MNC, (**D**) Degree, (**E**) EPC, (**F**) Bottleneck, (**G**) Eccentricity, (**H**) Closeness, (**I**) Radiality, (**J**) Betweenness, (**K**) Stress, and (**I**) Clustering Coefficient

On the basis of the PPI network, the critical subnetworks were extracted with Cytoscape software. The critical subnetworks and hub genes of the cervical lesions are shown in Fig. 9. The results showed that the hub genes constituted the key network of cervical carcinogenesis, and the genes were scored with the cytoHubba plug-in. Among the results of different algorithms, the common hub genes with high scoring were as follows: *NUSAP1*, *TOP2A*, *KIF2C*, *NDC80*, *ASPM*, *KIF20A*, *CDK1*, *KIF11*, *BIRC5*, *MCM2*, and *CHEK1*. Then, the MCODE plug-in was also used to analyze the PPI network, and 12 regions (subnetworks) closely related to the PPI network were found and separated. These regions might represent the molecular complex, and the results from the MCODE analysis included these high scoring hub genes and supported the results of the regional analysis (Table 2). These genes mainly affected cell division, the cell cycle, keratinocyte differentiation [32], DNA replication, mismatch repair, nucleoside exception repair, cytokine–cytokine receptor interaction, the chemokine signaling pathway and arachidonic acid metabolism during the progression of cervical cancer.

**Table 2:** Discovering regions closely related to the PPI network (MCODE)

Cluster	Score (Density*#Nodes)	Nodes	Edges	Genes
1	21.478	24	247	*TOP2A, KIF15, PRC1, SPAG5, KIF11, BIRC5, KIF23, MELK, DLGAP5, KIF2C, NUF2, MKI67, CENPE, NDC80, NEK2, AURKA, ASPM, CENPF, KIF20A, CDK1, TTK, CEP55, NUSAP1, KIF4A*
2	16.625	17	133	*RPTN, SPRR1B, SPRR2E, SPRR2D, LCE3D, DSC2, SPRR2B, DSG1, PI3, TGM1, CSTA, IVL, LOR, FLG, SPRR3, SPRR1A, PPL*
3	9.111	10	41	*CHEK1, MCM4, CDC45, MCM10, PRIM1, CDC7, CDT1, CDK2, POLE2, MCM8*
4	8	8	28	*CRISP3, PRSS3, HP, LRG1, CSTB, TCN1, TNFAIP6, CDA*
5	6	6	15	*SGOL2, CENPN, CASC5, CENPK, CDCA5, KNTC1*
6	6	6	15	*KRT78, KRT2, KRT13, KRT1, KRT16, KRT4*
7	6	6	15	*CXCR2, CXCR4, CXCL9, CXCL5, CXCL8, CXCL10*
8	6	6	15	*LY6G6C, CEACAM7, PSCA, CEACAM5, LYPD3, LY6D*
9	4	4	6	*RFC4, EXO1, TIPIN, RFC3*
10	4	4	6	*PRSS3P2, SERPINB3, FABP5, S100A7*
11	3.429	8	12	*DNA2, WDHD1, GINS2, DTL, RMI2, CDC6, BLM, MCM2*
12	3	3	3	*ALOX12B, ALOX12, ALOX15B*

### Expression and prognostic value of hub genes

**Fig. 10:**
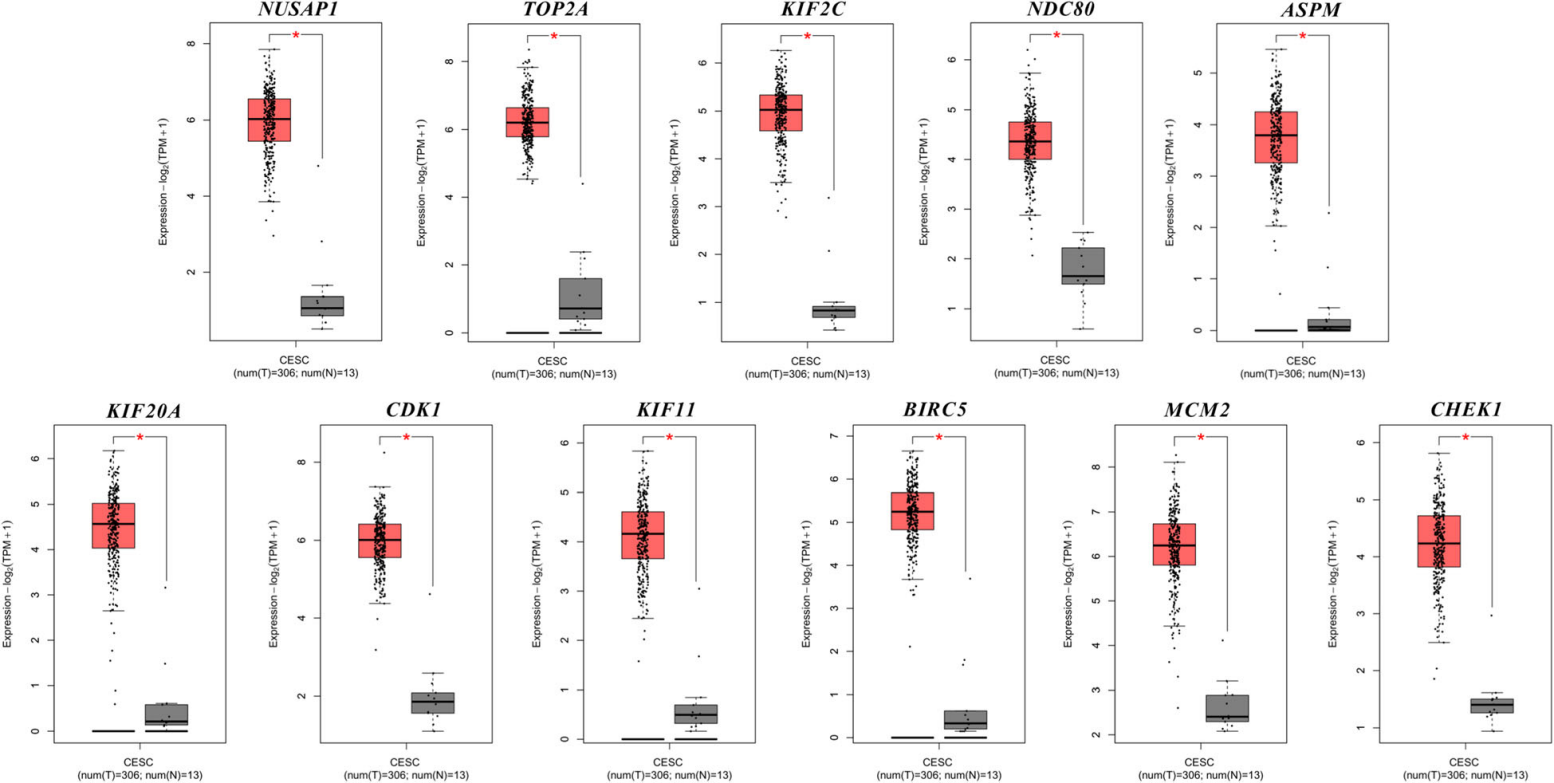
Expression boxplots of hub genes by GEPIA2. *NUSAP1, TOP2A, KIF2C, NDC80, ASPM, KIF20A, CDK1, KIF11, BIRC5, MCM2,* and *CHEK1* were significantly upregulated in cervical cancer tissues compared with normal tissues (*P* < 0.01)

**Fig. 11:**
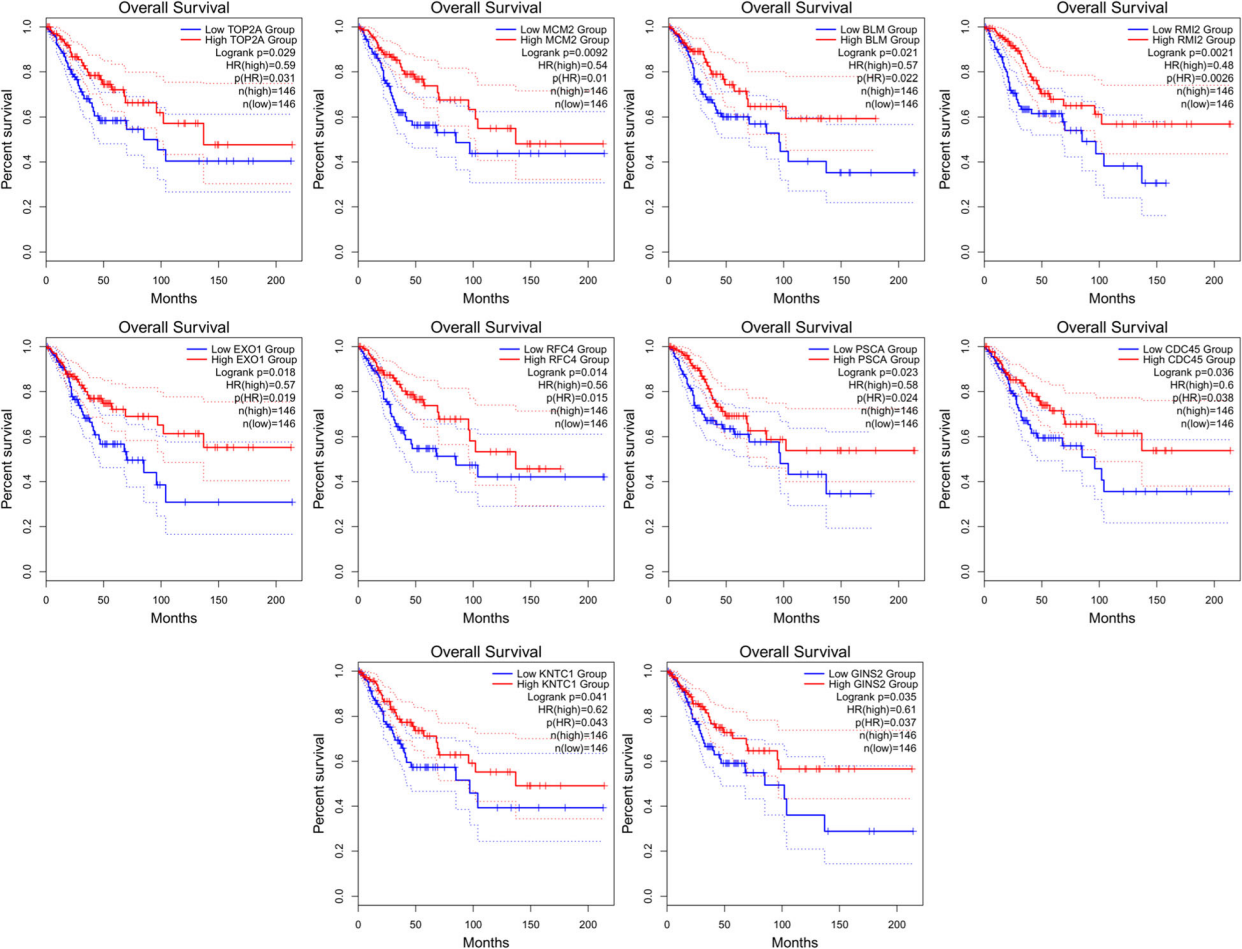
The expression of *MCM2, TOP2A, BLM, RMI2, EXO1, RFC4, PSCA, KNTC1, CDC45* and *GINS2* was significantly related to the overall survival of patients with CESC

**Fig. 12:**
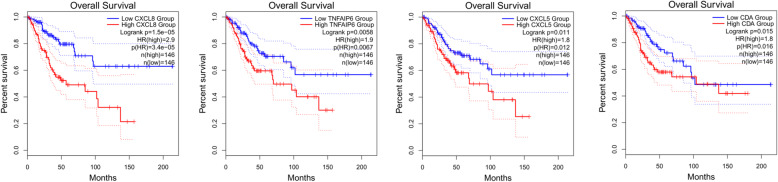
The expression of *CXCL8, TNFAIP6, CXCL5 and CDA* was significantly related to the overall survival of patients with CESC

GEPIA2 analysis showed that these hub genes were highly expressed in CESC tissues but weakly expressed in normal tissues (Fig. 10). Furthermore, to obtain more comprehensive prognostic information, hub genes from 12 subnetworks (Table 2) were subjected to survival analysis. Overall survival analysis by GEPIA2 indicated that the high expression of *MCM2*, *TOP2A*, *BLM*, *RMI2* [33], *EXO1*, *RFC4*, *PSCA*, *KNTC1*, *CDC45* and *GINS2* [34] in cervical lesions was correlated with an improved prognosis (Fig. 11). However, the high expression of *CXCL8* [35], *TNFAIP6*, *CXCL5* and *CDA* in cervical cancer tissues was associated with a poor patient prognosis (Fig. 12).

**Fig. 13:**
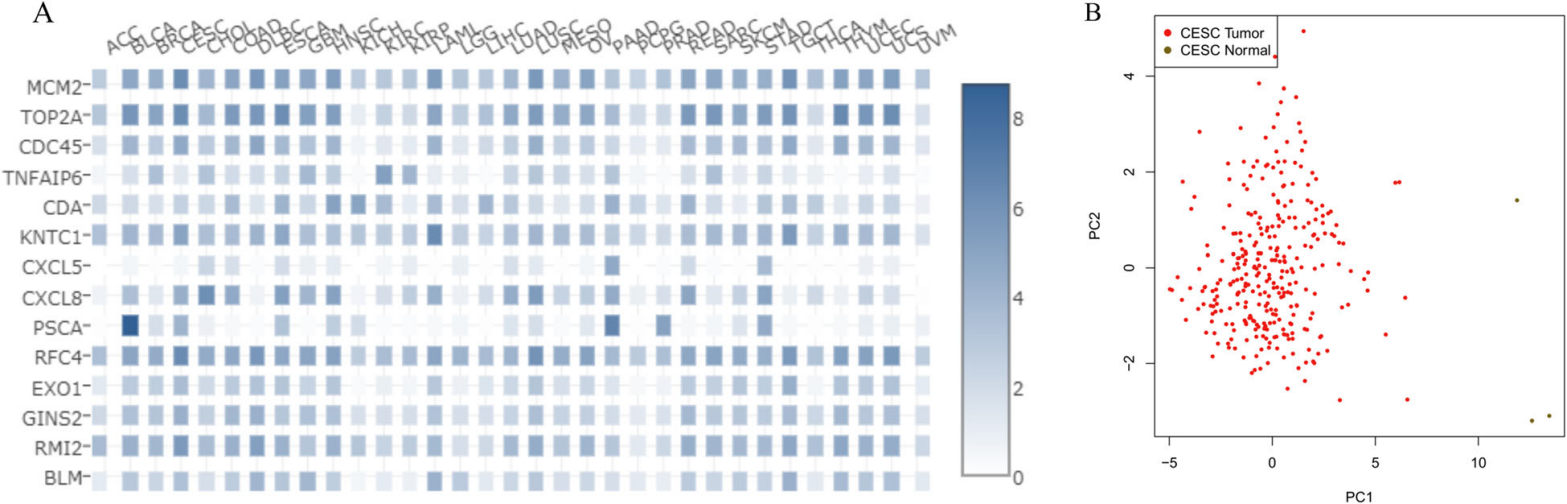
Multiple gene comparison and dimensionality reduction for prognosis. **A** Interactive heat map of tissue-specific expression in different cancer types. **B** Dimensionality reduction

**Fig. 14:**
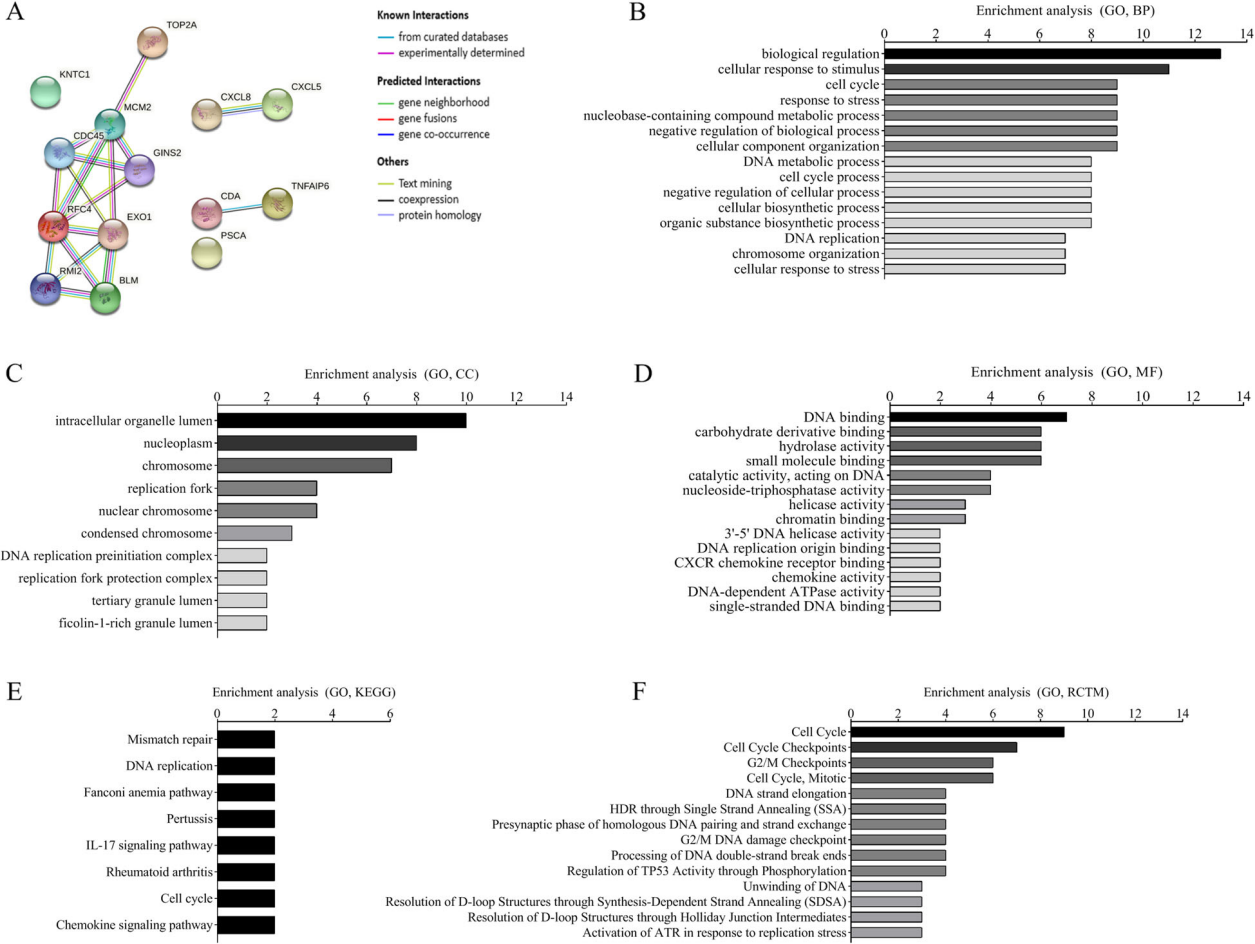
Key prognostic molecular interaction network model and enrichment analysis. **A** PPI network of key prognostic genes (PPI highest confidence 0.900). **B** Biological processes. **C** Cellular components. **D** Molecular functions. **E** KEGG pathways. **F** Reactome pathways

The tissue-specific expression of the hub genes in different cancer types is shown in Fig. 13 as an interactive heat map. A heat map was used to analyze the expression of the target genes in different tumor samples. Compared with other genes, *MCM2, TOP2A, CDC45, KNTC1, RFC4* and *RMI2* were highly expressed in CESC tumor tissues and might be better indicators of prognosis. PPI modeling (STRING) and enrichment analysis were performed on 14 target genes (Fig. 14). Gene functions according to the summaries of the Gene database (NCBI) are shown in Table 3. These findings might provide target genes for the prognosis and treatment of cervical cancer.

**Table 3:** Functional analysis of possible prognostic genes for the progression of cervical cancer

Gene	Function
*MCM2*	Minichromosome maintenance complex component 2. MCM2 is involved in the initiation of eukaryotic genome replication. It may be involved in the formation of replication forks and in the recruitment of other DNA replication-related proteins, and it regulates the helicase activity of the complex.
*TOP2A*	DNA topoisomerase II a. These findings might provide target genes for the prognosis and treatment of cervical carcinoma. TOP2A controls and alters the topologic state of DNA during transcription. This nuclear enzyme is involved in processes such as chromosome condensation, chromatid separation, and the relief of torsional stress that occurs during DNA transcription and replication.
*BLM*	BLM RecQ-like helicase. This Bloom-associated helicase unwinds a variety of DNA substrates including Holliday junctions and is involved in several pathways contributing to the maintenance of genome stability.
*RMI2*	RecQ-mediated genome instability 2. RMI2 plays a role in homologous recombination-dependent DNA repair and is essential for genome stability.
*EXO1*	Exonuclease 1. EXO1, with 5′ to 3′ exonuclease activity as well as RNase H activity, interacts with Msh2, which is involved in mismatch repair and recombination.
*RFC4*	Replication factor C subunit 4. The elongation of primed DNA templates by DNA polymerase delta and DNA polymerase epsilon requires the accessory proteins proliferating cell nuclear antigen (PCNA) and replication factor C (RFC).
*PSCA*	Prostate stem cell antigen. PSCA is highly expressed in the prostate and also expressed in the bladder, placenta, colon, kidney, and stomach. This gene is upregulated in a large proportion of prostate cancers and is also detected in cancers of the bladder and pancreas.
*KNTC1*	Kinetochore-associated 1. KNTC1 ensures proper chromosome segregation during cell division.
*CDC45*	Cell division cycle 45. CDC45, an essential protein required for the initiation of DNA replication, is important for the early steps of DNA replication in eukaryotes.
*GINS2*	GINS complex subunit 2. The GINS complex is essential for the initiation of DNA replication.
*CXCL8*	C-X-C motif chemokine ligand 8. CXC is a major mediator of the inflammatory response. This protein is also secreted by tumor cells and promotes tumor migration, invasion, angiogenesis and metastasis. This chemokine is also a potent angiogenic factor.
*TNFAIP6*	TNF alpha-induced protein 6. TNFAIP6 is a member of the hyaluronan-binding protein family. Its hyaluronan-binding domain is involved in extracellular matrix stability and cell migration. This protein is important in the protease network associated with inflammation.
*CXCL5*	C-X-C motif chemokine ligand 5. Chemokines, which recruit and activate leukocytes, promote angiogenesis and remodel connective tissues. It plays a role in cancer cell proliferation, migration, and invasion.
*CDA*	Cytidine deaminase. CDA is one of several deaminases responsible for maintaining the cellular pyrimidine pool.

## Discussion

Cervical cancer is one of the most common cancers among women worldwide. After persistent infection with high-risk HPV, the progression of inflammation to CIN to cancer often takes a long time. CIN is an important precancerous lesion of cervical cancer. At present, the prevention of cervical cancer depends mainly on HPV vaccines and HPV screening. Therefore, we need to not only prevent and monitor the development from normal cervical tissue or cells to CIN but also treat and block the development from CIN to cancer. Based on the analysis of multiple datasets, this study deepened our understanding of the molecular mechanism of cervical carcinogenesis and identified key prognostic genes. Potential biomarkers and target genes can be used to diagnose progressive disease before it leads to cancer.

In the present study, three expression profile datasets (GSE63514, GSE63217 and GSE138080) were downloaded from the GEO database. According to the development of cervical cancer, the samples were divided into three groups: N-CIN, CIN-CC and N-CC. Intersection analysis of these groups made the screening and validation of DEGs more reliable. A total of 476 DEGs were screened: 253 upregulated genes and 223 downregulated genes. These genes also regulate a number of biological pathways in the progression of cervical lesions, such as the cell cycle, DNA replication, rheumatoid arthritis, the p53 signaling pathway, bladder cancer, complement and coagulation cascades, amoebiasis, cytokine-cytokine receptor interaction, oocyte meiosis, and arachidonic acid metabolism. These pathways need to be further studied.

Furthermore, GSEA suggested that the DNA mismatch repair pathway plays an important role in the N-CIN process. Most importantly, the small cell lung cancer pathway might play an important role in the CIN-CC process. The cell cycle pathway might play an important role in the N-CC process. These results provide a better understanding of the molecular pathways associated with the development of the disease from normal cervical epithelium to CIN to cervical cancer. In the N-CIN stage, the enriched molecular pathways were DNA replication, nucleoside exception repair, DNA mismatch repair and specific amino acid metabolism (cysteine and methionine). With the development of cervical epithelial neoplasia, the enriched molecular pathways in the CIN-CC stage were small cell lung cancer, the adipocytokine signaling pathway, pathways in cancer, the Toll-like receptor signaling pathway, graft versus host disease and the TGF-beta signaling pathway, which play important roles in the occurrence and development of cancer. In the whole process of cervical lesions, we may need to pay close attention to pathways such as the cell cycle, DNA replication, DNA repair and pathways in cancer. Our results indicate the importance of these pathways in the occurrence and development of cervical cancer. We need to pay attention to the roles of these pathways in cervical carcinogenesis and further study their interactions.

Notably, the PPI network related to cervical lesions was composed of functional proteins that interacted with each other to participate in biological signal transmission, gene expression regulation, energy and material metabolism, cell cycle regulation and cell division. Eleven genes were identified as hub genes from 12 critical subnetworks of cervical cancer. Critical subnetworks might strongly contribute to the occurrence and development of cervical cancer and have high diagnostic value. Further analysis showed that *NUSAP1* [36]*, TOP2A, KIF2C* [37]*, NDC80* [23]*, ASPM* [21, 38]*, KIF20A* [39]*, CDK1* [19, 38]*, KIF11* [40, 41]*, BIRC5* [42]*, MCM2* and *CHEK1* [40, 41, 43] were high scoring hub genes and showed significantly upregulated expression in cervical cancer tissues compared with normal tissues (*P* < 0.01). By analyzing the prognostic values of the hub genes from the 12 subnetworks, a total of 14 potential molecular targets (*MCM2* [23, 35, 44–46], *TOP2A* [19, 35, 40], *BLM, RMI2*, *EXO1*, *RFC4*, *PSCA*, *KNTC1*, *CDC45*, *GINS2*, *CXCL8*, *TNFAIP6*, *CXCL5*, and *CDA*) were obtained and annotated.

These 23 key regulatory genes were enriched in different pathways, such as the cell cycle and mismatch repair, and play important roles in the occurrence and development of diseases. Our DAVID analysis showed that *CDK1*, *CHEK1*, *MCM2* and *CDC45* were involved in the cell cycle pathway. We found that the cell cycle pathway was very important to the progression of cervical cancer, which is worthy of further research. Mine KL et al. [47] proved that the cell cycle may be the main driving factor of cervical cancer. Van Dam et al. [48] showed that deregulation of the cell cycle is a major component of cervical cancer biology. Y Luo et al. [49] showed that *CDK1* might play an important role in regulating the genetic network related to the occurrence, development and metastasis of cervical cancer. Relevant studies also showed that the progression of cervical cancer can be affected by regulating the cell cycle, which highlights the biological significance of the cell cycle in cervical cancer [50–53]. In addition, Liang Zhao et al. [43] revealed that the imbalance of *CHEK1* and *CDKN2A* further promoted the proliferation of cancer cells by affecting the response of cell cycle checkpoints to DNA damage. Therefore, further research on the cell cycle and its related genes is of great significance. Moreover, we found that *CDK1* and *CHEK1* were involved in the p53 signaling pathway. The p53 signaling pathway is involved in the proliferation and apoptosis of cervical cancer cells and has high prognostic value.

The DNA replication pathway was also very important in the progression of cervical cancer in this study. Mitali Das et al. [54] showed that *MCM2–7* was significantly enriched in DNA replication, and high *MCM2–7* expression promoted the malignant proliferation of cervical cancer cells. *MCM2* was studied in a variety of human malignant tumors and was related to the histopathological grade of many [55–58]. Zheng J et al. [59] showed the diagnostic value of *MCM2* immunocytochemical staining in cervical lesions and its relationship with HPV infection. The expression of *RFC4* may also be associated with tumor progression and poor patient survival and is considered to be one of the main driving factors of the cervical cancer cell cycle network [47]. Dan Liu et al. [60] showed that *RFC4* was related to DNA replication and cell proliferation in cervical cancer. The abnormal expression of *RFC4* might be related to the progression of cervical cancer [61]. Similarly, our results indicated that *MCM2* and *RFC4* play an important role in the DNA replication pathway. In particular, *CXCL8* and *CXCL5* were important regulatory genes in our study. They are involved in rheumatoid arthritis and the cytokine–cytokine receptor interaction pathway in cervical carcinogenesis. CXCL8 was also associated with amoebiasis and the bladder cancer pathway. Ruiling Yan et al. [62] studied the clinical and prognostic value of *CXCL8* in patients with cervical cancer. The abnormal expression of *CXCL5* contributes to the tumorigenicity of cervical cancer [63]. Our results also showed that *RFC4* and *EXO1* are involved in DNA mismatch repair and are very important to cervical lesions.

However, due to the complexity of cervical cancer, its molecular mechanism needs to be further studied, and key regulatory genes and pathways will be continuously understood. We can track the influence of some key regulatory genes in cervical cancer through related research. For example, Beiwei Yu et al. [64] showed that *TOP2A* and *CENPF* are synergic master regulators that are activated in cancer. Jinhui Liu et al. [33] showed that *RMI2* was a novel key gene in CESC. Huan Chen et al. [65] showed that the *KNTC1* gene may be related to the pathophysiology of cervical cancer and may be one of the markers for the early diagnosis of cervical precancerous lesions. The abnormal expression of GINS2 can inhibit cell proliferation and tumorigenicity, as well as cell migration and invasion [66]. KIF20A expression is related to the overall survival rate of patients with early CESC and its progression [67]. At present, research on these key regulatory genes and related molecular pathways is lacking. The interactions of these genes and pathways in the progression of cervical cancer still need attention, research and verification.

Gene bioinformatics can provide a possible molecular targeting mechanism for the prevention and treatment of cervical diseases. Functional studies of candidate genes from public databases may lead to a better understanding of the development of cervical cancer. We divided the expression datasets into three groups according to the different stages of cervical lesions to comprehensively investigate the progression of cervical cancer. Intersection analysis of multiple expression profiles avoids the limitation of a single dataset and has repeatability and reliability. However, the limitation of this study was that the datasets were obtained from three different chip platforms, and as there were differences in data quality, the results were easily affected, resulting in bias. Our results may provide effective targets for the treatment of cervical cancer, but the effect on prognosis requires follow-up data, and further studies are needed to explore these key genes and important pathways.

## Conclusions

In conclusion, a comprehensive bioinformatics analysis of DEGs and pathways involved in the occurrence and development of cervical lesions was performed, and we explored and obtained key regulatory genes and pathways contributing to the progression of cervical cancer to improve prognosis. Moreover, these results may promote the understanding of molecular mechanisms and clinically related molecular targets for prognosis in cervical cancer and provide new insight into the occurrence and development of cervical cancer.

## Supplementary Information

**Additional file 1: **GO enrichment analysis of DEGs in Biological Processes, Cellular Components and Molecular Functions (DAVID). **Table S1.** GO enrichment analysis of DEGs in Biological Processes (DAVID). **Table S2.** GO enrichment analysis of DEGs in Cellular Components (DAVID). **Table S3.** GO enrichment analysis of DEGs in Molecular Functions (DAVID).

## Data Availability

The raw data of the three microarray datasets (accession numbers GSE63514, GSE64217 and GSE138080) were downloaded from the GEO repository (https://www.ncbi.nlm.nih.gov/geo/). All data are publicly accessible.

## Abbreviations

GEO: Gene Expression Omnibus
DEGs: Differentially expressed genes
GO: Gene Ontology
KEGG: Kyoto Encyclopedia of Genes and Genomes
GSEA: Gene set enrichment analysis
PPI: Protein–protein interaction
HPV: Human papillomavirus
CIN: Cervical intraepithelial neoplasia
N-CIN: From normal to cervical intraepithelial neoplasia
CIN-CC: From cervical intraepithelial neoplasia to cervical cancer
NCBI: National Center for Biotechnology Information
N-CC: From normal to cervical cancer
CESC: Cervical squamous cell carcinoma or endocervical adenocarcinoma
PCA: Principal component analysis
PCNA: Proliferating cell nuclear antigen
RFC: Replication factor C
