# Supplementary material for: Bioinformatics analysis of differentially expressed genes and pathways in the development of cervical cancer
Source: BMC Cancer. 2021 Jun 26;21:733. doi: 10.1186/s12885-021-08412-4 (PMC8236200; doi:10.1186/s12885-021-08412-4)
Supplement: Supplementary file 1 — Additional file 1: GO enrichment analysis of DEGs in Biological Processes, Cellular Components and Molecular Functions (DAVID). Table S1. GO enrichment analysis of DEGs in Biological Processes (DAVID). Table S2. GO enrichment analysis of DEGs in Cellular Components (DAVID). Table S3. GO enrichment analysis of DEGs in Molecular Functions (DAVID). [file 12885_2021_8412_MOESM1_ESM.docx]

**Table S1. GO enrichment analysis of DEGs in Biological Processes (DAVID)**

| Term | Count | *P-*value |
| --- | --- | --- |
| GO:0031424~keratinization | 16 | 6.46E-14 |
| GO:0006260~DNA replication | 24 | 5.16E-13 |
| GO:0018149~peptide cross-linking | 15 | 2.45E-12 |
| GO:0030216~keratinocyte differentiation | 17 | 7.89E-12 |
| GO:0007067~mitotic nuclear division | 28 | 9.13E-12 |
| GO:0008544~epidermis development | 17 | 4.78E-11 |
| GO:0000082~G1/S transition of mitotic cell cycle | 18 | 8.95E-11 |
| GO:0051301~cell division | 30 | 1.14E-09 |
| GO:0007059~chromosome segregation | 11 | 2.34E-06 |
| GO:0006270~DNA replication initiation | 8 | 5.17E-06 |
| GO:0006508~proteolysis | 29 | 7.15E-06 |
| GO:0000076~DNA replication checkpoint | 5 | 1.55E-05 |
| GO:0007062~sister chromatid cohesion | 12 | 1.75E-05 |
| GO:0061436~establishment of skin barrier | 6 | 3.51E-05 |
| GO:0008284~positive regulation of cell proliferation | 26 | 4.49E-05 |
| GO:0000083~regulation of transcription involved in G1/S transition of mitotic cell cycle | 6 | 1.26E-04 |
| GO:0000086~G2/M transition of mitotic cell cycle | 12 | 2.39E-04 |
| GO:0007051~spindle organization | 5 | 3.49E-04 |
| GO:0010951~negative regulation of endopeptidase activity | 11 | 3.61E-04 |
| GO:0000281~mitotic cytokinesis | 6 | 3.98E-04 |
| GO:0007018~microtubule-based movement | 9 | 4.23E-04 |
| GO:0010466~negative regulation of peptidase activity | 5 | 4.49E-04 |

**Table S2.GO enrichment analysis of DEGs in Cellular Components (DAVID)**

| Term | Count | *P*-value |
| --- | --- | --- |
| GO:0005615~extracellular space | 81 | 1.18E-16 |
| GO:0001533~cornified envelope | 16 | 2.91E-14 |
| GO:0070062~extracellular exosome | 120 | 2.26E-13 |
| GO:0005576~extracellular region | 79 | 1.89E-11 |
| GO:0000775~chromosome, centromeric region | 12 | 3.80E-08 |
| GO:0030496~midbody | 16 | 1.61E-07 |
| GO:0000777~condensed chromosome kinetochore | 12 | 3.19E-06 |
| GO:0005819~spindle | 13 | 1.48E-05 |
| GO:0005876~spindle microtubule | 8 | 4.50E-05 |
| GO:0005578~proteinaceous extracellular matrix | 18 | 9.82E-05 |
| GO:0005871~kinesin complex | 8 | 1.53E-04 |
| GO:0005737~cytoplasm | 148 | 2.76E-04 |
| GO:0031298~replication fork protection complex | 4 | 3.47E-04 |
| GO:0016324~apical plasma membrane | 17 | 7.62E-04 |
| GO:0005654~nucleoplasm | 84 | 0.002033688 |
| GO:0031012~extracellular matrix | 16 | 0.002447762 |

**Table S3. GO enrichment analysis of DEGs in Molecular Functions (DAVID)**

| Term | Count | *P*-value |
| --- | --- | --- |
| GO:0004252~serine-type endopeptidase activity | 23 | 5.44E-08 |
| GO:0008236~serine-type peptidase activity | 11 | 1.08E-06 |
| GO:0005198~structural molecule activity | 19 | 9.78E-06 |
| GO:0004867~serine-type endopeptidase inhibitor activity | 11 | 5.48E-05 |
| GO:0005125~cytokine activity | 14 | 1.46E-04 |
| GO:0016887~ATPase activity | 14 | 2.16E-04 |
| GO:0003777~microtubule motor activity | 9 | 3.75E-04 |
| GO:0008017~microtubule binding | 14 | 7.35E-04 |
| GO:0004869~cysteine-type endopeptidase inhibitor activity | 6 | 8.32E-04 |
